# A General Framework for 3-D Parameters Estimation of Roads Using GPS, OSM and DEM Data

**DOI:** 10.3390/s18010041

**Published:** 2017-12-25

**Authors:** Christophe Boucher, Jean-Charles Noyer

**Affiliations:** Laboratoire d’Informatique Signal et Image de la Côte d’Opale, Université du Littoral Côte d’Opale, 59183 Dunkerque, France; jean-charles.noyer@univ-littoral.fr

**Keywords:** multi-sensor fusion, non-linear filtering, GNSS-based navigation, land-vehicle localization, digital road maps, digital elevation models

## Abstract

A growing number of applications needs GIS mapping information and commercial 3-D roadmaps especially. This paper presents a solution of accessing freely to 3-D map information and updating in the context of transport applications. The method relies on the OSM road networks that is 2-D modeled intrinsically. The objective is to estimate the road elevation and inclination parameters by fusing GPS, OSM and DEM data through a nonlinear filter. An experimental framework, using ASTER GDEM2 data, shows some results of the improvement of the roads modeling that includes their slopes also. The map database can be enriched with the estimated inclinations. The accuracy depends on the GPS and DEM elevation errors (typically a few meters with the GNSS sensors used and the DEM under consideration).

## 1. Introduction

Nowadays, an increasing number of transport applications would need to have access to 3-D map information whether for location or route planning problems [1]. Some solutions of 3-D maps exist but they are commercial and expensive. In this context, the purpose of our work is to offer a free solution based on the use of OpenStreetMap (OSM, http://www.openstreetmap.org). OSM is a free web-service that provides map data from uploaded GPS traces and user-registered information as names, types, traffic directions and inclinations of roads [2]. More generally, OSM is built by volunteer users who contribute and maintain these GIS data all over the world. The level of accuracy depends primarily on existing data. It should be noticed that the level of accuracy is optimal when one is in a dense area for that many users have refined the data over time. Providing none 3-D support, the OSM database is increasingly used in the development of transport applications [3,4]. In our earlier work [5], a method has been proposed to update the OSM roads slopes by using a car positioning system composed of 2 GPS units. To make the method more robust, we are interested in the fusion of the dual-GPS positioning system and data from a Digital Elevation Model (DEM) that accounts for the terrain relief. This can lead to the improvement of the estimated elevations [6]. One of the objectives of the Advanced Spaceborne Thermal Emission and Reflection Radiometer (ASTER) mission was to produce a global DEM of a very large part of the Earth, based on a horizontal resolution of 1 arcsecond (30 m) [7]. In 2009, this American-Japanese collaboration allows to produce a model whose horizontal resolution was three times sharper than the DEM of the Shuttle Radar Topography Mission (SRTM) [8] that was built with at 3 arcseconds (about 90 m). However, the presence of many voids and artifacts in this first model version made the exploitation of data quite difficult. In 2011, a new model version was proposed with a significant refinement of the relief. Some artifacts as steps at scene boundaries, pits, bumps or mole runs are fixed without deleting all of them. Therefore, 1 × 1 degree tiles of data are available with additional information of the elevation measurements quality. The WGS84 horizontal datum is used to locate ASTER data while the EGM96 vertical datum levels the stored elevations compared to the Mean Sea Level (MSL) geoid. Several authors published works about the relevance and the accuracy of DEM data in comparison with calibrated GPS-altitudes [9,10]. Considering the horizontal resolution of ASTER DEM data, an elevation information is available about every 30 m. This low spatial sampling can be a major drawback of the ASTER data. In a process of estimating the slope of roads, it is hard to achieve a high level of accuracy using ASTER DEM only [11]. The possibility to use DEM only solutions is indeed related to the spatial dimensions of the tiles that are for now not small enough. Three-dimensional modeling of road networks has resulted in many publications of methods based on the processing of aerial images [12], geometric methods using 2D GIS data only [13], or methods based on the fusion of GPS/GIS/DEM data [14,15,16,17].

This paper proposes to enrich the modeling of road network with an additional parameter related to the inclination and the vertical height of roads. By fusing GPS/DEM data through a nonlinear filter (typically an unscented Kalman filter [18]), our method allows to get an estimation of the OSM roads slopes. The aim is to complete the mapping system with 3-D information as nodes elevations and inclinations of roads. We equipped a standard vehicle with a Global Navigation Satellite System (GNSS) based on a dual-GPS system whose receivers antennas are aligned on the extremities of the same side of the roof. A such consideration induces a more accurate positioning of the vehicle by bounding the possible errors due to GPS signal degradation [19]. Knowing that OSM provides a digital modeling of roads that relies of the 2-D geolocation of their nodes depending on land surveys, the method requires a map-matching step with GPS data. The goal is to deliver an estimation of the road inclinations. To this end, we develop an original method based on a geometric approach to manage the DEM data and compute a more accurate MSL-elevation. In the proposed solution, all measurements are sequentially processed by an unscented Kalman filter (UKF) that is more suited to nonlinear equations than the standard Kalman filter. An original aspect of our approach lies in the modeling of the OSM and DEM databases. They are considered as sensors with their own measurement errors which makes possible to develop a global filtering approach. Finally, by injecting the estimated vehicle elevations and their orthogonal projections on the matched road into a least squares method, we get an estimate of the slope of this road.

We have chosen to validate this approach in a context of road experimentation mainly composed of highways that are built in an open environment with a varied but limited terrain elevation. We present experimental results of 3-D time-location of the vehicle that allow to get an accurate estimation of the inclination of the roads traveled during the test. Then, it becomes possible to register them in OSM for later use in 3-D route planning applications, as developed in [20].

## 2. 3-D Modeling

Stating the standard kinematic equations are enough to model the car dynamics, the major parameters are defined by the acceleration γ, the velocity *v* and the position. They are reported in the following state vector by introducing their *x*, *y* and *z*-coordinates along the 3-D axes:

(1) $$X_{t}={\left(x_{t},\;v_{t}^{x},\;\gamma_{t}^{x},\;y_{t},\;v_{t}^{y},\;\gamma_{t}^{y},\;z_{t},\;v_{t}^{z},\;\gamma_{t}^{z}\right)}^{T}$$

### 2.1. Formatting of the Dynamics Equation

Introducing the *u*-axis with u={x,y,z}, the car dynamics relies on the following third order kinematic state model:

(2) $$\left(\begin{array}{c} u_{t+1}\\ v_{t+1}^{u}\\ \gamma_{t+1}^{u} \end{array}\right)=\underset{F_{u}}{\underset{︸}{\left(\begin{array}{ccc} 1 & \Delta t & 0\\ 0 & 1 & \Delta t\\ 0 & 0 & 1 \end{array}\right)}}\left(\begin{array}{c} u_{t}\\ v_{t}^{u}\\ \gamma_{t}^{u} \end{array}\right)+\left(\begin{array}{c} \Omega_{t}^{u}\\ \Omega_{t}^{v^{u}}\\ \Omega_{t}^{\gamma^{u}} \end{array}\right)$$

The dynamics matrix $F_{u}$ reveals the navigation rate Δt. The Ω-vector is composed of additive white Gaussian noises with $\Omega_{t}∼N\left(0,Q_{t}\right)$. This equation can be generalized to the three-dimensional case and becomes:

(3) $$X_{t+1}=f\left(X_{t},\Omega_{t}\right)=FX_{t}+\Omega_{t}$$

*F* models the linear flow of the dynamics. Stating $O_{3\times 3}$, the null square-matrix of dimension 3, it can be expanded as:

(4) $$F=\left(\begin{array}{ccc} F_{x} & O_{3\times 3} & O_{3\times 3}\\ O_{3\times 3} & F_{y} & O_{3\times 3}\\ O_{3\times 3} & O_{3\times 3} & F_{z} \end{array}\right)$$

### 2.2. Formatting the Measurement Equations

#### 2.2.1. GPS Readings

The car carries two EGNOS-aided (SBAS) receivers that collect the GPS positioning (the specifications of the GPS sensors are given in Section 5.1). Their antennas are fixed on the roof and are spaced by a distance *D*. $Z_{t+1}^{\mathrm{GPS},i}$ denotes the measurement that is providing by the receiver i={1,2}. Both them are fused sequentially to get an estimation of the 3-D dynamics:

(5) $$Z_{t+1}^{\mathrm{GPS},i}=\left(\begin{array}{c} x_{t+1}^{\mathrm{GPS},i}\\ y_{t+1}^{\mathrm{GPS},i}\\ z_{t+1}^{\mathrm{GPS},i} \end{array}\right)=h^{\mathrm{GPS},i}\left(X_{t+1}\right)+V_{t+1}^{\mathrm{GPS},i}$$

$V_{t+1}^{\mathrm{GPS},i}∼N\left(0,R_{t+1}^{\mathrm{GPS}}\right)$ is an additive white Gaussian noise with zero mean and $R_{t+1}^{\mathrm{GPS}}$ as the noise covariance matrix.

A single coordinate system is used to define the car positioning. It refers to the GPS antenna of unit #1. Taking into account this consideration, the measurement function of each GPS unit can be formatted as follows:

- for the GPS unit #1, $h^{\mathrm{GPS},1}$ is the linear measurement function:
  (6) $$h^{\mathrm{GPS},1}\left(X_{t+1}\right)=\left(\begin{array}{c} x_{t+1}\\ y_{t+1}\\ z_{t+1} \end{array}\right)$$
- for the GPS unit #2, $h^{\mathrm{GPS},2}$ is the nonlinear measurement function:
  (7) $$h^{\mathrm{GPS},2}\left(X_{t+1}\right)=\left(\begin{array}{c} x_{t+1}+D*\frac{v_{t+1}^{x}}{∥v_{t+1}∥}\\ y_{t+1}+D*\frac{v_{t+1}^{y}}{∥v_{t+1}∥}\\ z_{t+1}+D*\frac{v_{t+1}^{z}}{∥v_{t+1}∥} \end{array}\right)$$

Here, the introduced non-linearities are derived from the calculations making it possible to define the GPS measurement $Z_{t+1}^{\mathrm{GPS},2}$ in the coordinate system that is fitted to the antenna of the receiver #1.

#### 2.2.2. OSM Road Network

The next step is to fuse the previous GPS-estimated parameters with the OSM database in order to match the traveled road. As a consequence, the accuracy of the car ground-positioning can be increased since the OSM road network is modeled by road segments whose 2-D location of transitional nodes from one segment to another is available. The accuracy of the GPS-based positioning is straightforwardly related to the intrinsic performance of the GPS receiver (e.g., about a few meters for our sensors). Fusing GPS-based estimation with the OSM data allows to consider the road geometry as a constraint for the car displacement. In this case, the estimator can take benefit of the accuracy of the map database. The ground-location $\left(x_{t+1}^{\mathrm{OSM}},y_{t+1}^{\mathrm{OSM}}\right)$ and the ground-direction of its road segment $\theta_{t+1}^{\mathrm{OSM}}$ are the main features of each node:

(8) $$Z_{t+1}^{\mathrm{OSM}}=\left(\begin{array}{c} x_{t+1}^{\mathrm{OSM}}\\ y_{t+1}^{\mathrm{OSM}}\\ \theta_{t+1}^{\mathrm{OSM}} \end{array}\right)=h^{\mathrm{OSM}}\left(X_{t+1}\right)+V_{t+1}^{\mathrm{OSM}}$$

$V_{t+1}^{\mathrm{OSM}}$ is an additive white Gaussian noise with zero mean and $R_{t+1}^{\mathrm{OSM}}$ as the noise covariance matrix so that the statistical aspects related to the inhomogeneities and errors of land surveys can be integrated. In order to link the stater vector components to the measurements, a new nonlinear function $h^{\mathrm{OSM}}$ is introduced as:

(9) $$h^{\mathrm{OSM}}\left(X_{t+1}\right)=\left(\begin{array}{c} x_{t+1}\\ y_{t+1}\\ \arctan\left(\frac{v_{t+1}^{y}}{v_{t+1}^{x}}\right) \end{array}\right)$$

When the vehicle is stopped, the previous direction is reused.

#### 2.2.3. ASTER GDEM2 Terrain Surface

The final step is the identification of the DEM-area in which the car is driven. Once matched the MSL-elevation is fused with the previous OSM-estimated parameters by the proposed algorithm that takes into account the ASTER GDEM2 horizontal resolution of 1 arcsecond (about 30m). The DEM measure features the ground-location of the MSL-elevation point as $\left(x_{t+1}^{\mathrm{DEM}},y_{t+1}^{\mathrm{DEM}},z_{t+1}^{\mathrm{DEM}}\right)$.

(10) $$Z_{t+1}^{\mathrm{DEM}}=\left(\begin{array}{c} x_{t+1}^{\mathrm{DEM}}\\ y_{t+1}^{\mathrm{DEM}}\\ z_{t+1}^{\mathrm{DEM}} \end{array}\right)=h^{\mathrm{DEM}}\left(X_{t+1}\right)+V_{t+1}^{\mathrm{DEM}}$$

$h^{\mathrm{DEM}}$ is the measurement function and $V_{t+1}^{\mathrm{DEM}}$ is an additive white Gaussian noise with zero mean and $R_{t+1}^{\mathrm{DEM}}$ as the noise covariance matrix. This matrix defines the standard deviations relative to the horizontal position and vertical elevation measurement.

(11) $$h^{\mathrm{DEM}}\left(X_{t+1}\right)=\left(\begin{array}{c} x_{t+1}\\ y_{t+1}\\ z_{t+1}-H \end{array}\right)$$

This function highlights the term *H* that defines the height that has been measured initially between GPS antenna of the unit #1 and the ground.

## 3. Estimation of the Road Inclination

**Figure 1:**
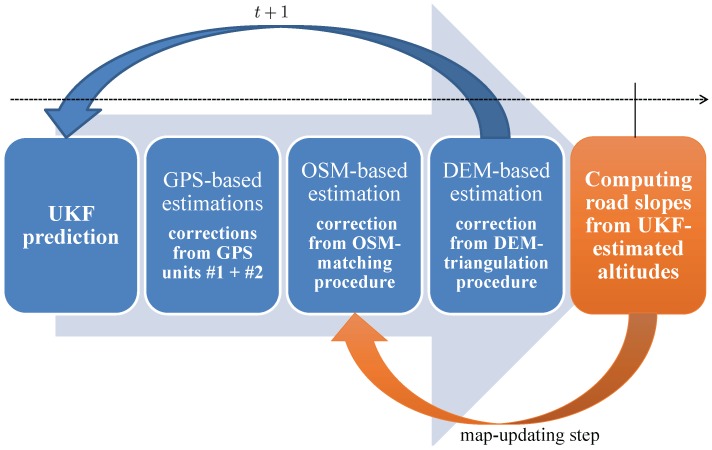
Multisensor fusion structure.

The goal of this paper is to propose a theoretical framework to OSM data enrichment with road inclination. The proposed nonlinear modeling is solved by an unscented Kalman filter (UKF). It is known that the UKF approach improves the state estimation compared with the EKF-methods in the nonlinear case [21]. The benefit of the UKF-approach also lies in its lower computational complexity compared with the particle filtering methods [22] which are not essential here due to moderate non-linearity of the modeling. Figure 1 shows that the filter is based on a sequential processing structure of the GPS, OSM and DEM data. Finally, the filter estimates the inclination which is used to enrich the road network database.

### 3.1. Dynamics Sampling: Prediction

A first step consists in a statistical sampling of the state-space vector using sigma-points. Let $\chi_{t|t}^{j,X}$ and $\chi_{t|t}^{j,\Omega }$ be the sigma-points at time step *t* based on the knowledge of the measurement at time step *t*, respectively related to the state vector *X* and the dynamics noise Ω.

Their evolution is computed as follows:

(12) $$\chi_{t+1|t}^{j,X}=f\left(\chi_{t|t}^{j,X},\chi_{t|t}^{j,\Omega }\right)$$

$\chi_{t+1|t}^{j,X}$ is the $j^{\mathrm{th}}$ column of $\chi_{t+1|t}^{X}$. The predicted state is computed as follows:

(13) $${\hat{X}}_{t+1|t}=\sum_{j=1}^{2n_{a}+1}w_{j}\chi_{t+1|t}^{j,X}$$

$n_{a}$ is the augmented state vector dimension and the weights $w_{j}$ are derived from the unscented transform [23]. The covariance matrix ${\tilde{P}}_{t+1|t}$ is written as below:

(14) $${\tilde{P}}_{t+1|t}=\sum_{j=1}^{2n_{a}+1}w_{j}\left(\chi_{t+1|t}^{j,X}-{\hat{X}}_{t+1|t}\right){\left(\chi_{t+1|t}^{j,X}-{\hat{X}}_{t+1|t}\right)}^{T}$$

### 3.2. Correction: General Principles

Let us denote $Z^{\alpha }$ the measurement related to the sensor α where $\alpha \in \{{\mathrm{GPS}}_{1},{\mathrm{GPS}}_{2}$, OSM, DEM}. The measurement equation can be written as:

(15) $$Z_{t+1}^{\alpha }=h^{\alpha }\left(X_{t}\right)+V_{t+1}^{\alpha }$$

From a general viewpoint, the correction step use the sigma-points $\chi_{t+1|t}^{j,Z^{\alpha }}$ related to the measurement of the sensor α:

(16) $$\chi_{t+1|t}^{Z^{\alpha }}=h^{\alpha }\left(\chi_{t+1|t}^{X}\right)+\chi_{t+1|t}^{V^{\alpha }}$$

The predicted measurement ${\hat{Z}}_{t+1|t}^{\alpha }$ is computed as the weighted sum of the sigma-points $\chi_{t+1|t}^{j,Z^{\alpha }}$ where the superscript *j* denotes the $j^{\mathrm{th}}$ column of $\chi_{t+1|t}^{Z^{\alpha }}$:

(17) $${\hat{Z}}_{t+1|t}^{\alpha }=\sum_{j=1}^{2n_{a}+1}w_{j}\chi_{t+1|t}^{j,Z^{\alpha }}$$

The state vector and the error covariance matrix are computed as follows:

(18) $$\left\{\begin{array}{cc} K_{t+1}^{\alpha } & ={\tilde{P}}_{X_{t+1}Z_{t+1}^{\alpha }}{\tilde{P}}_{Z_{t+1}^{\alpha }Z_{t+1}^{\alpha }}^{-1}\\ {\tilde{P}}_{t+1|t+1}^{\alpha } & ={\tilde{P}}_{t+1|t}-K_{t+1}^{\alpha }{\tilde{P}}_{Z_{t+1}^{\alpha }Z_{t+1}^{\alpha }}{K_{t+1}^{\alpha }}^{T}\\ {\hat{X}}_{t+1|t+1}^{\alpha } & ={\hat{X}}_{t+1|t}+K_{t+1}^{\alpha }{(Z}_{t+1}^{\alpha }-{\hat{Z}}_{t+1|t}^{\alpha }) \end{array}\right.$$

where

(19) $$\begin{array}{cc} {\tilde{P}}_{X_{t+1}Z_{t+1}^{\alpha }} & =\sum_{j=1}^{2n_{a}+1}w_{j}\left(\chi_{t+1|t}^{j,X}-{\hat{X}}_{t+1|t}\right){\left(\chi_{t+1|t}^{j,Z^{\alpha }}-{\hat{Z}}_{t+1|t}^{\alpha }\right)}^{T} \end{array}$$

(20) $$\begin{array}{cc} {\tilde{P}}_{Z_{t+1}^{\alpha }Z_{t+1}^{\alpha }} & =\sum_{j=1}^{2n_{a}+1}w_{j}\left(\chi_{t+1|t}^{j,Z^{\alpha }}-{\hat{Z}}_{t+1|t}^{\alpha }\right){\left(\chi_{t+1|t}^{j,Z^{\alpha }}-{\hat{Z}}_{t+1|t}^{\alpha }\right)}^{T} \end{array}$$

### 3.3. Correction: Proposed Solution

The proposed solution is based on a sequential processing of sensor data. An original feature of our method consists in a statistical modeling of map data from OSM and DEM. The popular approaches match the estimate of the position with the road network [24]. Our statistical method allows to take into account the mapping inaccuracies in the estimation process. Indeed, in many solutions, the map matching procedure uses a projection onto the road network and thus does not consider the map accuracy as a parameter of the problem. Our approach uses this accuracy to build a map-matching procedure that gives in the estimation process a relative weight to the OSM-based correction. This is done via the measurement covariance matrix $R_{t}^{\mathrm{OSM}}$. The accuracy of the OSM measurements is relative to the accuracy of the GPS traces uploaded by the users in the database.

Moreover, the DEM database, also modeled as an additional sensor, is used to correct the estimation of the elevation of the vehicle. Similarly to OSM measurements, the DEM data are used in the estimation process, considering their accuracy defined by the covariance matrix $R_{t}^{\mathrm{DEM}}$. Again with this ”sensor”, we do not use a all-or-nothing correction but a weighted correction using the DEM measurement covariance matrix. This leads to an improvement of the estimation accuracy, especially of the elevation which remains a parameter with high errors in GPS (typically several meters in some cases or more in multipath propagation problems of GNSS signals).

As a consequence, the integration of OSM and DEM data leads to a better estimation of the road network parameters and in particular of the road sections elevation (including slope). This estimation is performed in real time and improves the OSM database by extending it to the 3-D case. The updated database will then be used for future positioning thus leading to an overall improvement in estimation.

Figure 1 details the different steps of the filtering solution. The measurements are sequentially processed in the following order: GPS readings #1 and #2, OSM measurements and DEM measurements. The relative weight of each sensor is related to their measurement error covariance matrix which initially appears in the computation of the sigma-points $\chi_{t+1|t}^{Z^{\alpha }}$ related to measurement equation Equation (16).

#### 3.3.1. Correction from GPS Sensors

The GPS readings of each receiver #*i* (i={1,2}) are sequentially processed to estimate the state vector ${\hat{X}}_{t+1/t+1}^{{\mathrm{GPS}}_{i}}$.

#### 3.3.2. Correction from the OSM Database

The OSM database is modeled as an additional sensor (see Equation (16)). The prediction of the map measurement ${\hat{Z}}_{t+1|t+1}^{\mathrm{OSM}}$ is done from the unscented transform (Equation (17)). This prediction is matched with the OSM road network and the matched segment is used to correct the estimation of the ground-location and direction of the vehicle.

In [24], the author lists various approaches for map-aided positioning of a vehicle and most of them concerns geometric methods. We propose to solve the map matching step through a statistical approach that relies on the computing of the Mahalanobis distance [25] between the UKF-predicted map measurement and all available OSM road descriptors. Knowing that the digital road map is described by geolocated and interconnected nodes whose main drawback is their random sampling grid, we have refined the map modeling. In the matching step, we introduce as inputs the orthogonal projections of the UKF-predicted map measurement onto each nearby OSM road segment if possible, otherwise the nearest line segment extremity is used instead of the projection. This step leads to the identification of the position of the closest candidate for each road segment of the map database.

At time instant t+1, we get the set of orthogonal projections $Z_{m}^{\mathrm{OSM}}$:

(21) $$Z_{m}^{\mathrm{OSM}}={\mathrm{proj}}_{⊥,m}\left({\hat{Z}}_{t+1|t+1}^{\mathrm{OSM}}\right)$$

m={1,…,n} and *n* describes the maximum projections number. Map descriptor includes the road direction. So, the Mahalanobis distance $d_{m}=d\left(Z_{m}^{\mathrm{OSM}},{\hat{Z}}_{t+1|t+1}^{\mathrm{OSM}}\right)$ can be evaluated in the cases of a two-way road $\left(\theta_{1},\theta_{2}\right)$ or a one-way road $\left(\theta_{1}\right)$:

(22) $$\left\{\begin{array}{c} Z_{m}^{\mathrm{OSM},\theta_{1}}={\left(x_{m}^{\mathrm{OSM}},\;y_{m}^{\mathrm{OSM}},\;\theta_{m}^{\mathrm{OSM}}\right)}^{T}\\ Z_{m}^{\mathrm{OSM},\theta_{2}}={\left(x_{m}^{\mathrm{OSM}},\;y_{m}^{\mathrm{OSM}},\;\theta_{m}^{\mathrm{OSM}}+\pi \right)}^{T} \end{array}\right.$$

The Mahalanobis distances can be derived from the previous equation:

(23) $${\left(d_{m}^{\theta_{k}}\right)}^{2}={\left(Z_{m}^{\mathrm{OSM},\theta_{k}}-{\hat{Z}}_{t+1|t+1}^{\mathrm{OSM}}\right)}^{T}{\left({\tilde{P}}_{t+1|t+1}^{\mathrm{GPS}}\right)}^{-1}\left(Z_{m}^{\mathrm{OSM},\theta_{k}}-{\hat{Z}}_{t+1|t+1}^{\mathrm{OSM}}\right)\;\mathrm{with}\;k=\{1,2\}$$

The innovation process has a Gaussian distribution. As a consequence, the squared Mahalanobis distance is then distributed according to a chi-square law with three degrees of freedom. The correction is done if the value is below a threshold ε that results from the statistical properties of the Mahalanobis distance. Indeed, the measurement $Z_{m^{*}}^{\mathrm{OSM},\theta^{*}}$ that minimizes the criterion $d_{m}^{\theta }\left(Z_{m}^{\mathrm{OSM}},{\hat{Z}}_{t+1|t+1}^{\mathrm{OSM}}\right)\leq \varepsilon$ is used to correct the GPS-based estimate.

#### 3.3.3. Correction from DEM Database

A DEM database is used to take into account the elevation in the multisensor processing. A first solution has been proposed in [26] and was based on the minimization of Mahalanobis distances between the nearest horizontal point of elevation and the filter estimation. In area of low elevation change, this approach may be sufficient. In areas with higher elevation, the use of raw DEM data can generate significant errors. In Section 4, we develop our main contribution that is relative to a new modeling of DEM data based on triangulations.

The last step concerns the correction with elevation measurements from the ASTER terrain surface. The goal is to bound with DEM corrections [27], the well-known altitude errors of GPS measurements [28]. The position and elevation errors of the DEM data are modeled by Equation (10) and the unscented transform is used to derive the predicted measurement. It must be noticed that the elevation components only are corrected to avoid horizontal positioning errors that can be caused by the poor horizontal resolution of the DEM. So, the measurement $Z_{0}^{\mathrm{DEM}}$ is used to correct the previous estimate and deliver the final estimate:

(24) $${\hat{X}}_{t+1|t+1}={\hat{X}}_{t+1|t+1}^{\mathrm{DEM}}$$

### 3.4. Road Slope Computing

The final objective of this work is to propose an enrichment of OSM database with the inclinations of the roads. This step is done from the estimated elevation (*z*-component of the state vector in Equation (24)) and the matched orthogonal projection on the OSM road network (see Equation (21)). The last step relies then on the estimation of the inclination parameters.

The following equation details the relationship between the elevation $E_{s,t}$ and the radial distance $\rho_{s,t}$ of the projected estimation at *t* onto the line segment #*s* to the extremity $\left(x_{s,0},y_{s,0}\right)$:

(25) $$E_{s,t}=a_{s}\rho_{s,t}+z_{s,0}$$

where $a_{s}$ denotes the slope of the line segment #*s* and $z_{s,0}$ its intercept.

For the $n_{s}$ projections onto the same line segment #*s*, Equation (25) can be written as follows:

(26) $$E_{s}=Hu=\left(\begin{array}{cc} \rho_{s,1} & 1\\ \vdots & \vdots \\ \rho_{s,n_{s}} & 1 \end{array}\right)\left(\begin{array}{c} a_{s}\\ z_{s,0} \end{array}\right)$$

where the radial distance $\rho_{s,t}$ is described by:

(27) $$\rho_{s,t}=\sqrt{{\left(x_{s,t}^{\mathrm{OSM}}-x_{s,0}\right)}^{2}+{\left(y_{s,t}^{\mathrm{OSM}}-y_{s,0}\right)}^{2}}\;,\;\forall t$$

H is a $n_{s}\times 2$ matrix. The parameter $n_{s}$ changes for each line segment #*s* and depends on several parameters (vehicle speed, length of the line segment, …).

For each segment, a least squares method is used to estimate the road inclination:

(28) $$\left(\begin{array}{c} {\hat{a}}_{s}\\ {\hat{z}}_{s,0} \end{array}\right)={\left(H^{T}H\right)}^{-1}H^{T}E_{s}$$

This statistical estimation of the inclination is used to automatically enrich the road map database.

## 4. New Modeling of DEM Data

We propose a new modeling of DEM-data based on the triangulations in the vicinity of the ground-location of the vehicle, instead of matching raw data as published in [26]. So, the problem is now to estimate the elevation between two successive grid points. This method allows to compute the pseudo-elevation of the vehicle from the DEM grid. Here, the new advantage is that the DEM management method takes into account the topology of all terrain.

### 4.1. Triangulation of the DEM

Another solution can be to decompose the grid into triangles. We use the rasterized DEM to produce a triangulated irregular network (TIN). This modeling produce a continuous surface that can be used in the filtering solution in a similar way to OSM road network (which is a line segment network). This modeling avoids the problem of estimating planes equations using a least squares method which can lead to difficulties in high variability of the orientation of the plane (for an approach to 4 points) and introduce additional errors in calculating the elevation mainly due to the calculation method. The advantage of a triangle-based approach is that it does not generate any additional errors.

**Figure 2:**
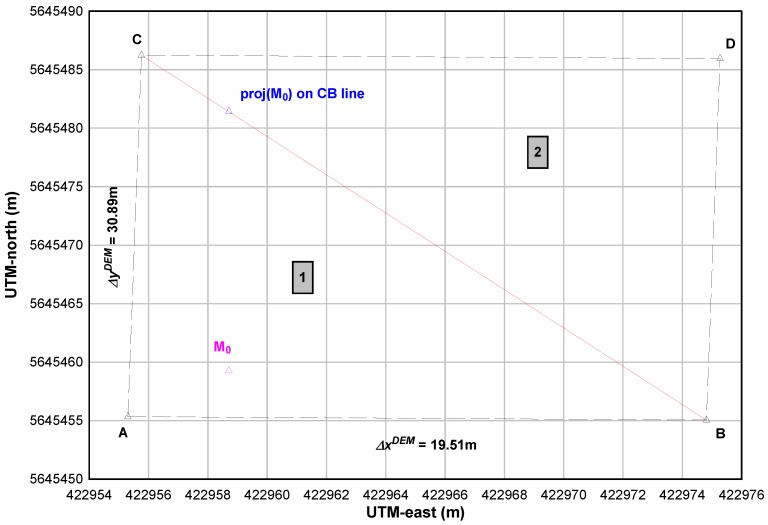
Example of triangulation of the ASTER GDEM2 grid.

Figure 2 shows a part of the ASTER GDEM2, for instance. $A={\left(x_{i}^{\mathrm{DEM}},\;y_{i}^{\mathrm{DEM}},\;z_{ij}^{\mathrm{DEM}}\right)}^{T}$ is the 2-D grid point (i,j) for which the MSL-elevation $z_{ij}^{\mathrm{DEM}}$ is known.

The triangles #1 (ABC) and #2 (BCD) are defined by these additional points:

- $B={\left(x_{i}^{\mathrm{DEM}}+\Delta x^{\mathrm{DEM}},\;y_{i}^{\mathrm{DEM}},\;z_{i+1,j}^{\mathrm{DEM}}\right)}^{T}$
- $C={\left(x_{i}^{\mathrm{DEM}},\;y_{i}^{\mathrm{DEM}}+\Delta y^{\mathrm{DEM}},\;z_{i,j+1}^{\mathrm{DEM}}\right)}^{T}$
- $D={\left(x_{i}^{\mathrm{DEM}}+\Delta x^{\mathrm{DEM}},\;y_{i}^{\mathrm{DEM}}+\Delta y^{\mathrm{DEM}},\;z_{i+1,j+1}^{\mathrm{DEM}}\right)}^{T}$

The fixed horizontal resolution of the grid allows to write:

(29) $$\left\{\begin{array}{c} x_{i+1}^{\mathrm{DEM}}=x_{i}^{\mathrm{DEM}}+\Delta x^{\mathrm{DEM}}\\ y_{i+1}^{\mathrm{DEM}}=y_{i}^{\mathrm{DEM}}+\Delta y^{\mathrm{DEM}} \end{array}\right.$$

The plane equation based on the triangle #1 needs to compute the normal $\vec{n_{1}}$ to the (ABC) plane:

(30) $$\vec{n_{1}}=\vec{AB}\times \vec{AC}=\left(\begin{array}{c} -\Delta y^{\mathrm{DEM}}\left(z_{i+1,j}^{\mathrm{DEM}}-z_{i,j}^{\mathrm{DEM}}\right)\\ -\Delta x^{\mathrm{DEM}}\left(z_{i,j+1}^{\mathrm{DEM}}-z_{i,j}^{\mathrm{DEM}}\right)\\ \Delta x^{\mathrm{DEM}}\Delta y^{\mathrm{DEM}} \end{array}\right)$$

Then, the condition for a point $M={\left(x,\;y,\;z\right)}^{T}$ to be on the plane (ABC) is written as follows:

(31) $$\vec{AM}\cdot \vec{n_{1}}=0$$

This leads to the equation of the plane (ABC) that supports the triangle #1:

(32) $$z=z_{i,j}^{\mathrm{DEM}}+\frac{z_{i+1,j}^{\mathrm{DEM}}-z_{i,j}^{\mathrm{DEM}}}{\Delta x^{\mathrm{DEM}}}\left(x-x_{i}^{\mathrm{DEM}}\right)+\frac{z_{i,j+1}^{\mathrm{DEM}}-z_{i,j}^{\mathrm{DEM}}}{\Delta y^{\mathrm{DEM}}}\left(y-y_{i}^{\mathrm{DEM}}\right)$$

The point *A* being the support point, this equation gives the vertical elevation of the point *M* as a function of these horizontal coordinates that is the goal to reach in the proposed method.

The point *D* is now the support point and the plane equation based on the triangle #2 needs to compute the normal $\vec{n_{2}}$ to the (BCD) plane:

(33) $$\vec{n_{2}}=\vec{DB}\times \vec{DC}=\left(\begin{array}{c} \Delta y^{\mathrm{DEM}}\left(z_{i+1,j+1}^{\mathrm{DEM}}-z_{i,j+1}^{\mathrm{DEM}}\right)\\ \Delta x^{\mathrm{DEM}}\left(z_{i+1,j+1}^{\mathrm{DEM}}-z_{i+1,j}^{\mathrm{DEM}}\right)\\ -\Delta x^{\mathrm{DEM}}\Delta y^{\mathrm{DEM}} \end{array}\right)$$

The point $M={\left(x,\;y,\;z\right)}^{T}$ belongs to the plane (BCD) if:

(34) $$\vec{DM}\cdot \vec{n_{2}}=0$$

The plane equation of (BCD) that supports the triangle #2 is:

(35) $$\begin{array}{ccc} z & = & z_{i+1,j+1}^{\mathrm{DEM}}+\frac{z_{i+1,j+1}^{\mathrm{DEM}}-z_{i,j+1}^{\mathrm{DEM}}}{\Delta x^{\mathrm{DEM}}}\left(x-x_{i+1}^{\mathrm{DEM}}\right)\\ & & +\frac{z_{i+1,j+1}^{\mathrm{DEM}}-z_{i+1,j}^{\mathrm{DEM}}}{\Delta y^{\mathrm{DEM}}}\left(y-y_{i+1}^{\mathrm{DEM}}\right) \end{array}$$

So, Equations (32) and (35) allow to compute the vertical elevation at the point *M* that belongs to the triangle #1 or #2.

### 4.2. Identification of the Relevant Triangle and Computation of the MSL-Elevation

The challenge is now to identify the relevant triangle of the point *M*. At this step, the last estimator allows to fix the horizontal location of the point in the DEM by writing:

(36) $$\left\{\begin{array}{c} x=x_{t+1}^{\mathrm{OSM}}\\ y=y_{t+1}^{\mathrm{OSM}} \end{array}\right.$$

To compute the elevation of the point *M*, it must be determined if the point $M_{0}^{\mathrm{DEM}}={\left(x_{0}^{\mathrm{DEM}}=x,y_{0}^{\mathrm{DEM}}=y,z_{0}^{\mathrm{OSM}}\neq z\right)}^{T}$ belongs to the triangle (ABC) or (BCD). For this, one computes the sum of the angles formed by the point $M_{0}$ and the tops of the relevant triangle:

(37) $$\left\{\begin{array}{c} S_{\left(ABC\right)}=\hat{AM_{0}B}+\hat{BM_{0}C}+\hat{CM_{0}A}\\ S_{\left(BCD\right)}=\hat{BM_{0}C}+\hat{CM_{0}D}+\hat{DM_{0}B} \end{array}\right.$$

If $S_{\left(ABC\right)}=2\pi$ the relevant triangle is #1, otherwise $S_{\left(BCD\right)}=2\pi$ and the relevant triangle is #2. From Equations (32) or (35), the elevation $z_{0}^{\mathrm{DEM}}$ will be computed to lead to the following measurement that is used in the DEM-correction step (see part Section 3.3.3):

(38) $$Z_{0}^{\mathrm{DEM}}=M_{0}^{\mathrm{DEM}}$$

## 5. Experimental Framework

A scenario has been defined to validate our method. It relies on available OSM and ASTER GDEM2 data in a selected area (3 km by 3 km) of the city of Calais, France. We focus here on the results of estimated roads inclinations.

### 5.1. Scenario Context

**Figure 3:**
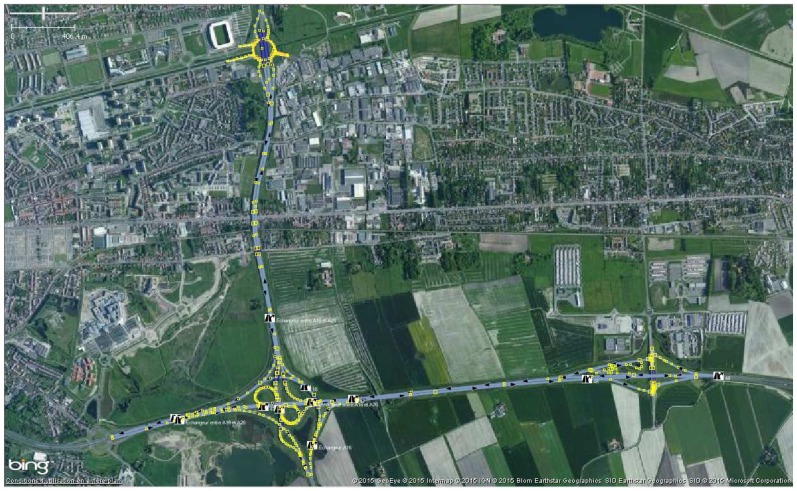
Aerial view of the OSM road network.

The objective is to estimate the roads inclinations. Theses roads are extracted from the OSM database. The experimentation context is shown in Figure 3. A car is driven on this part of OSM road network composed of motorways mainly. It is fitted with a dual-GPS system that relies on two u-blox EVK-6T receivers that collect the 3-D vehicle positionings. The receiver manufacturer announces the position accuracy as 2.5 m CEP (Circular Error Probable) and 2 m CEP if SBAS is enabled. These precisions may be more or less degraded depending on the experimental conditions (multipaths, etc.).

The GPS antennas are located on the car roof and aligned with the driver side. The spacing distance is D= 1.75 m. Their height from ground is H= 1.55 m. The improved SBAS positioning (EGNOS) has been enabled on each receiver whose GPS navigation rate has been set to 1 Hz.

**Figure 4:**
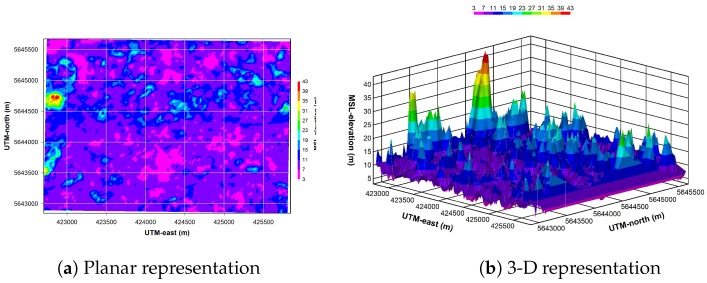
ASTER V2 elevation levels in the vicinity of the OSM road network.

The ASTER GDEM2 informs on the elevation levels of the experimentation area. They are plotted in Figure 4a,b. This dataset is composed of a grid of 14,560 MSL-elevation points that are spaced by a known horizontal distance: $\Delta x^{\mathrm{DEM}}=\Delta y^{\mathrm{DEM}}=$ 1 arcsecond (or 30 m) in a LLH geographical datum; these values must be recomputed if a horizontal UTM datum is used. In addition, it must be noticed that some voids and artifacts (step, pit-in-bump, mole-run, etc.) remain in this version of dataset, although they announced as decreased in the northern area or almost disappeared. The ASTER GDEM2 is provided with a quality parameter that informs on the measurement accuracy of the elevation. This information can be used in the matching procedure of DEM to reject the measure, if needed.

Note that the u-blox GPS receivers directly deliver MSL-elevations in the EGM84 vertical datum while the ASTER GDEM V2 use the geoid of EGM96. The prerequisite is also to convert the u-blox MSL-elevations in the same vertical datum. This is done by using the MSP GEOTRANS software [29].

### 5.2. Map-Matching Procedure

**Figure 5:**
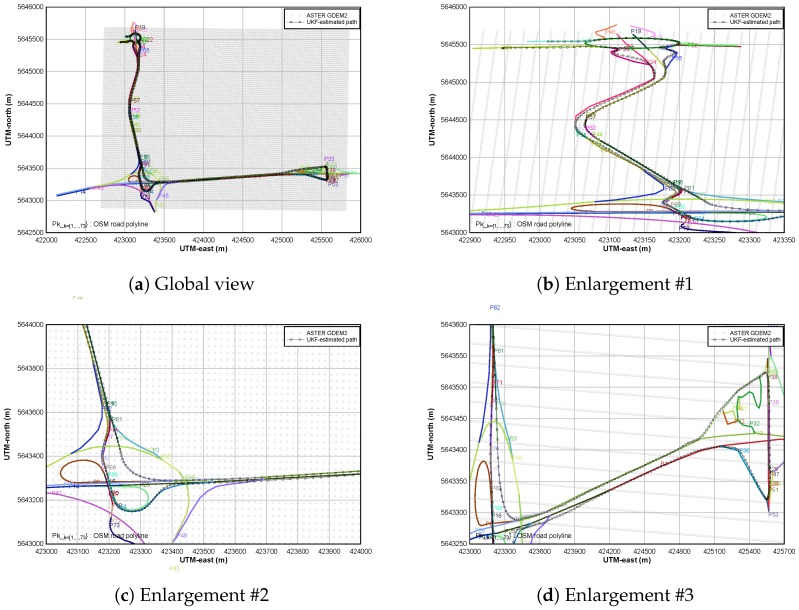
Estimated path of the vehicle vs. OSM road network.

Our OSM dataset is composed of 73 polylines that represent various roads as primary or secondary roads, motorways, etc. These polylines are modeled by nodes and road segments whose total number is 417 as shown in Figure 5a. The 2-D location of MSL-elevation points are also plotted (light grey) on this figure. The main objective is here to estimate the slope of each road segment on which the car is driven. Therefore, the UKF-based algoritm allows to realize the fusion of GPS/OSM/ASTER data for estimating the car path that is plotted in black squares here. Selected enlargements of the estimated path are available in Figure 5b–d.

**Figure 6:**
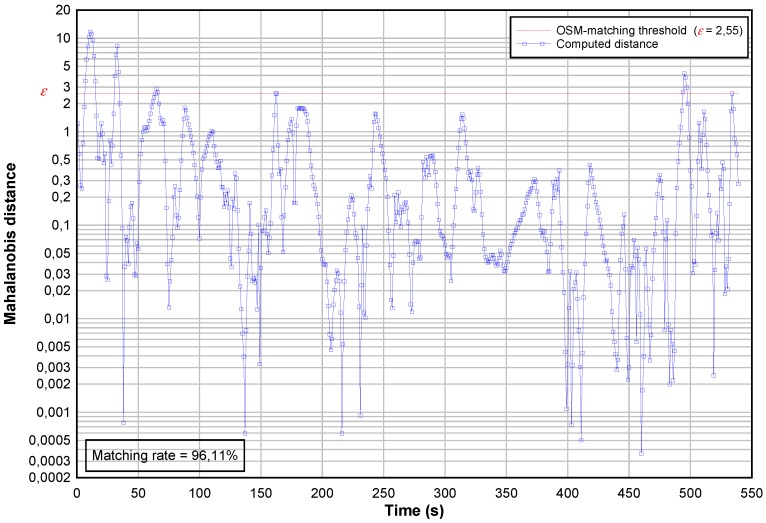
Time-evolution of the OSM-based matching criterion.

Once the two GPS positioning fused, the OSM-matching step allows to identify the road segment on which the car is located. This procedure relies on the computing of Mahalanobis distances whose the time-evolution is plotted on Figure 6, highlighting ε that is the OSM-matching threshold. In order to refine also the ground-location of the vehicle, this threshold is used to decide when the OSM road network can be used. The map-matching step reaches a rate of 96.30%, knowing that a 100% score is difficult to get due to land surveying errors or missing roads.

### 5.3. Road Inclination Estimation

**Figure 7:**
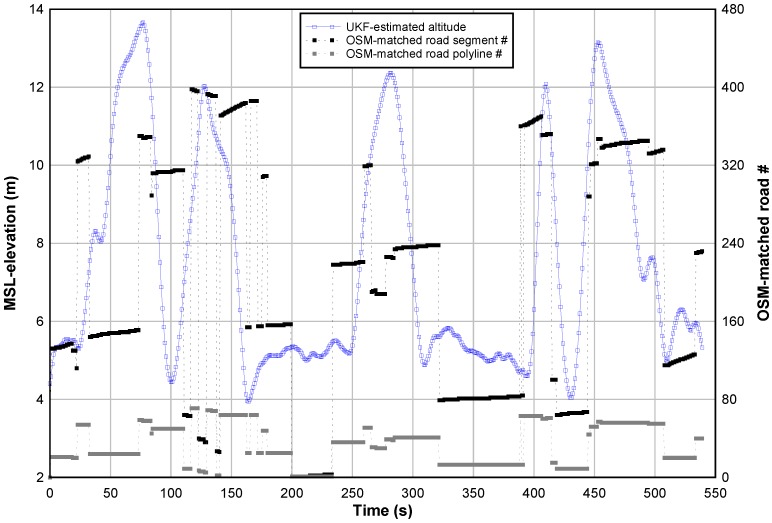
UKF-estimated altitudes vs. OSM matched roads.

The proposed approach fuses the GPS-based altitudes and the matched ASTER-based elevations to refine the estimated altitude of the car as shown in Figure 7 where the time-evolution is plotted in blue circles. This figure shows also the number of all matched OSM road segments—during the defined scenario—that are plotted in black squares. To estimate the slope of each road segment, the proposed least squares method needs the UKF-estimated altitudes and the matched map data as input.

**Figure 8:**
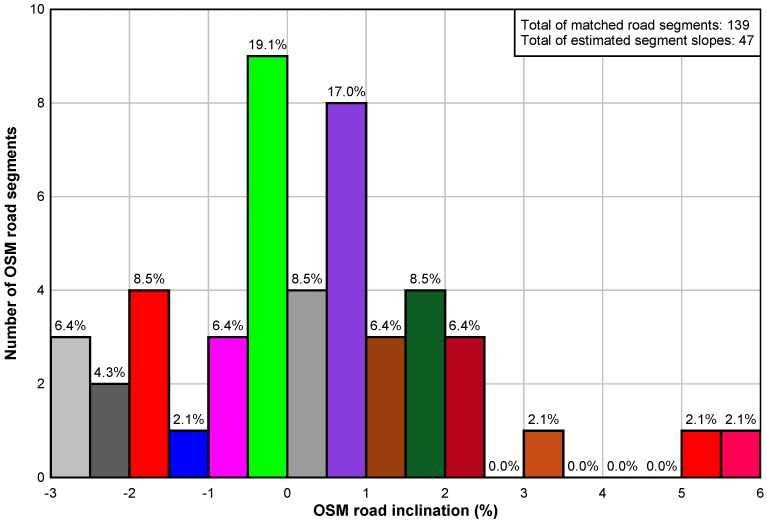
Estimation of the OSM road inclinations.

As detailed in Equation (25), the road segment needs to be traveled during 2 GPS time-instants to estimate the inclination but in order to improve the estimation, the minimum number of needed GPS readings is set to 4. Figure 8 shows the results of roads inclination percentage. They concern only the OSM road segments that the car could traveled during the test scenario. Overall, 34% of traveled road segments have benefited from an estimate of their inclination. Among these estimations, about 28% of these road segments are in an area with rather flat terrain. It is possible to find an explanation in heavy climbs or descents when the vehicle has taken the entrances or exits of motorways that have undergone an elevation to their construction. The vehicle has also used exchangers that are generally on an upward or downward slope. Note that the estimation accuracy depends to the minimum number of available UKF-estimated altitudes and this parameter can be set dynamically in term of road segment length, car speed and GPS navigation rate.

## 6. Conclusions

This work states a general approach to estimate the inclinations of OSM road segments. It relies on the collected data from a dual-GPS positioning, an OSM road network and an ASTER GDEM2 terrain surface that are fused following a centralized scheme. The proposed method takes into account the topology of the geographical area and allows a new computation of the elevation point from the terrain models. The map-matching procedure allows to select the used OSM road segments and the triangulation of DEM leads to a refinement of the ASTER MSL-elevation. Therefore, it becomes possible to enhance the OSM database with the estimated slope of each matched road segment automatically. Then, the road inclination parameter is available for further more reliable route planning applications, for instance those of electric vehicles whose autonomy is yet limited for trips. Based on the OSM concept, our method is designed for a collaborative enhancement of the database. This general and theoretical framework is suitable to the implementation of other kinds of GNSS, DEM, etc. It provides the basis for a 3-D modeling of the road segments in a map database. Cases of GPS outages and non-existing OSM roads are not considered in this work. The next step is now to study multi-GNSS positioning systems and alternative roadmap databases.
